# Supplementary material for: Sex Chromosome Turnover in Bent-Toed Geckos (Cyrtodactylus)
Source: Genes (Basel). 2021 Jan 19;12(1):116. doi: 10.3390/genes12010116 (PMC7832896; doi:10.3390/genes12010116)
Supplement: Supplementary file 1 [file genes-12-00116-s001.pdf]

Supplemental Information

Supplemental Table 1: List of *Cyrtodactylus chaunghanakwaensis* and *C. pharbaungensis* specimens used for RADseq and PCR.

Species	ID	Sex	Locality
Cyrtodactylus chaunghanakwaensis	LSUHC13294	female	16.18456°N, 97.98814°E
Cyrtodactylus chaunghanakwaensis	LSUHC13296	female	16.18456°N, 97.98814°E
Cyrtodactylus chaunghanakwaensis	LSUHC13297	female	16.18456°N, 97.98814°E
Cyrtodactylus chaunghanakwaensis	LSUHC13299	female	16.18456°N, 97.98814°E
Cyrtodactylus chaunghanakwaensis	LSUHC13301	female	16.18456°N, 97.98814°E
Cyrtodactylus chaunghanakwaensis	LSUHC13303	female	16.18456°N, 97.98814°E
Cyrtodactylus chaunghanakwaensis	LSUHC13304	female	16.18456°N, 97.98814°E
Cyrtodactylus chaunghanakwaensis	LSUHC13306	female	16.18456°N, 97.98814°E
Cyrtodactylus chaunghanakwaensis	LSUHC13307	female	16.18456°N, 97.98814°E
Cyrtodactylus chaunghanakwaensis	LSUHC13309	female	16.18456°N, 97.98814°E
Cyrtodactylus chaunghanakwaensis	LSUHC13310	female	16.18456°N, 97.98814°E
Cyrtodactylus chaunghanakwaensis	LSUHC13295	male	16.18456°N, 97.98814°E
Cyrtodactylus chaunghanakwaensis	LSUHC13298	male	16.18456°N, 97.98814°E
Cyrtodactylus chaunghanakwaensis	LSUHC13300	male	16.18456°N, 97.98814°E
Cyrtodactylus chaunghanakwaensis	LSUHC13302	male	16.18456°N, 97.98814°E
Cyrtodactylus chaunghanakwaensis	LSUHC13305	male	16.18456°N, 97.98814°E
Cyrtodactylus chaunghanakwaensis	LSUHC13308	male	16.18456°N, 97.98814°E
Cyrtodactylus chaunghanakwaensis	LSUHC13312	male	16.18456°N, 97.98814°E
Cyrtodactylus chaunghanakwaensis	LSUHC13313	male	16.18456°N, 97.98814°E
Cyrtodactylus chaunghanakwaensis	LSUHC13314	male	16.18456°N, 97.98814°E
Cyrtodactylus pharbaugensis	LSUHC13373	female	16.22866°N, 97,94,236°E
Cyrtodactylus pharbaugensis	LSUHC13374	female	16.22866°N, 97,94,236°E
Cyrtodactylus pharbaugensis	LSUHC13377	female	16.22866°N, 97,94,236°E
Cyrtodactylus pharbaugensis	LSUHC13378	female	16.22866°N, 97,94,236°E
Cyrtodactylus pharbaugensis	LSUHC13380	female	16.22866°N, 97,94,236°E

Cyrtodactylus pharbaugensis	LSUHC13384	female	16.22866°N, 97,94,236°E
Cyrtodactylus pharbaugensis	LSUHC13385	female	16.22866°N, 97,94,236°E
Cyrtodactylus pharbaugensis	LSUHC13389	female	16.22866°N, 97,94,236°E
Cyrtodactylus pharbaugensis	LSUHC13390	female	16.22866°N, 97,94,236°E
Cyrtodactylus pharbaugensis	LSUHC13371	male	16.22866°N, 97,94,236°E
Cyrtodactylus pharbaugensis	LSUHC13372	male	16.22866°N, 97,94,236°E
Cyrtodactylus pharbaugensis	LSUHC13375	male	16.22866°N, 97,94,236°E
Cyrtodactylus pharbaugensis	LSUHC13376	male	16.22866°N, 97,94,236°E)
Cyrtodactylus pharbaugensis	LSUHC13379	male	16.22866°N, 97,94,236°E
Cyrtodactylus pharbaugensis	LSUHC13381	male	16.22866°N, 97,94,236°E
Cyrtodactylus pharbaugensis	LSUHC13382	male	16.22866°N, 97,94,236°E
Cyrtodactylus pharbaugensis	LSUHC13383	male	16.22866°N, 97,94,236°E
Cyrtodactylus pharbaugensis	LSUHC13386	male	16.22866°N, 97,94,236°E
Cyrtodactylus pharbaugensis	LSUHC13387	male	16.22866°N, 97,94,236°E
Cyrtodactylus pharbaugensis	LSUHC13388	male	16.22866°N, 97,94,236°E

Supplemental Table 2: List of specimens, voucher ID, and GenBank Accession Number for the ND2 sequence data.

**Species**	**Voucher**	**GenBank Number**
Agamura persica	MNH 247474	JX440515
Alsophylax pipiens	CAS 238804	KC151973
Altiphylax stoliczkai	CES 10/1237	KJ794404
Crossobamon orientalis	ID 7607	KC151975
Cyrtodactylus adorus	Cyad PR1	HQ401165
Cyrtodactylus aequalis	LSUHC 12895	MF872275
Cyrtodactylus agusanensis	KU 320009	HQ154529
Geckoella albofasciatus	CES 09/1117	KM878625
Cyrtopodion aravallensis	CES 13/1458	KJ794406
Cyrtopodion mansarulus	CES 11/1319	KJ794414
Cyrtopodion scabrum	TG 109	JX041345
Dravidogecko janakiae	BNHS2357	MN520268
Hemidactylus aaronbaueri	CES14023	MN482222
Hemidactylus acanthopholis	CES 14009	MG711530
Hemidactylus albofasciatus	BNHS 1579	EU268378
Hemidactylus anamallensis	ZSIK 2969	MN520264
Hemidactylus angulatus	EBG 746	HM559620
Hemidactylus ansorgii	ZFMK 87671	MN843756
Hemidactylus aquilonius	CAS 228109	EU268374
Hemidactylus bayonii	CAS 263352	MN843764
Hemidactylus benguellensis	CAS 258530	MN843788
Hemidactylus bowringii	CAS 206649	EU268373
Hemidactylus brasilianus	MZUSP 92493	EU268351
Hemidactylus brookii	CAS 229632	GQ458051
Hemidactylus cf. gleadowi	CES11014	MH454761
Hemidactylus cf. kushmorensis	CES11065	MH454765
Hemidactylus craspedotus	LSHC 5613	HM559618
Hemidactylus depressus	AMB 7481	HM559625
Hemidactylus fasciatus	CAS 207777	EU268371
Hemidactylus flaviviridis	KUZ R72238	AB937992
Hemidactylus frenatus	LLG 4871	GQ458049
Hemidactylus garnotii	CAS 223286	EU268363
Hemidactylus giganteus	JB 03	HM559632
Hemidactylus gracilis	BNHS 1592	EU268379
Hemidactylus graniticolus	CES15257	MH454763
Hemidactylus greeffi	CAS 219044	EU268369
Hemidactylus haitianus	AMB 4188	HM559633

Hemidactylus hemchandrai	CES16279	MH454764
Hemidactylus hunae	AMB 7416	HM559640
Hemidactylus imbricatus	TG 00568	EU268354
Hemidactylus kangerensis	CES14184	MK569823
Hemidactylus karenorum	CAS 210670	EU268362
Hemidactylus lankae	AMB 7453	HM559648
Hemidactylus leschenaultii	AMB 7443	HM559635
Hemidactylus longicephalus	CAS 218939	HM559637
Hemidactylus mabouia	YPM 14798	HM559639
Hemidactylus macropholis	CAS 227520	JX041369
Hemidactylus maculatus	BNHS 1516	HM559641
Hemidactylus malcolmsmithi	IAG061	MK569842
Hemidactylus mercatorius	AMNH R- 16825	MN843845
Hemidactylus muriceus	MVZ 249709	MN843848
Hemidactylus murrayi	CAS 206638	GQ458054
Hemidactylus nzingae	CAS 258424	MN843864
Hemidactylus paivae	CAS 263311	MN843873
Hemidactylus palaichthus	LSUMZ H–12421	EU268368
Hemidactylus parvimaculatus	ADS36	GQ458053
Hemidactylus persicus	CAS 227612	EU268377
Hemidactylus platyurus	KU 304111	HM559619
Hemidactylus prashadi	JB 30	HM559643
Hemidactylus reticulatus	CES13062	MH454767
Hemidactylus robustus	MVZ 248437	EU268376
Hemidactylus sahgali	JB 08	HM559650
Hemidactylus sataraensis	CES14220	MH454768
Hemidactylus scabriceps	CES12008	MH454769
Hemidactylus subtriedrus	JB noID	HM627909
Hemidactylus sushilduttai	CES11079	MN482236
Hemidactylus tenkatei	USNM 579728	KM975945
Hemidactylus treutleri	CES14116	MN482237
Hemidactylus triedrus	NCBS AU703	MH666065
Hemidactylus turcicus	LSU1981	EU268360
Hemidactylus vanam	BNHS 2329	MG711532
Hemidactylus whitakeri	NCBS-AU713	MH666066
Hemidactylus yajurvedi	CES12006	MH454772
Mediodactylus russowii	JX440517	JX440517
Stenodactylus slevini	MAM 3066	KC151986
Stenodactylus yemenensis	MVZ 236566	HQ443548
Tenuidactylus caspius	CAS 228602	KC151988

Tropiocolotes confusus	TMHC406	MG990926
Tropiocolotes nubicus	JB123	KC151991
Tropiocolotes scorteccii	CN8984	MG990930
Tropiocolotes somalicus	TMHC455	MG990931
Tropiocolotes steudneri	JB 28	JX440520
Cyrtodactylus Dayang		USNMHC_2396
Cyrtodactylus Mizoram	CES13/1455	KM255197
Cyrtodactylus adorus	Cyad.SH2	KT363927
Cyrtodactylus aequalis	LSUHC 12895	MF872275
Cyrtodactylus agusanensis	KU 320016	HQ154532
Cyrtodactylus albofasciatus	CES09/1117	KM878625
Cyrtodactylus angularis	CUMZ<THA>:R:2005.07.30.138	GU550717
Cyrtodactylus annandalei	CAS 215722	JX440524
Cyrtodactylus annulatus	KU 314945	JX678807
Cyrtodactylus arcanus	AMS R124559	JQ820314
Cyrtodactylus astrum	LSUHC 10024	JX519472
Cyrtodactylus aunglini	LSUHC 13948	MH764589
Cyrtodactylus auralensis	LSUHC 7346	KT013127
Cyrtodactylus aurensis	LSUHC 7286 NADH	JX440525
Cyrtodactylus auribalteatus	CyAuNRZ001	AP018116
Cyrtodactylus australotitiwangsaensis	LSUHC 6637	JX519484
Cyrtodactylus ayeyarwadyensis	CAS 212459	JX440526
Cyrtodactylus badenensis	Tr 0001	MT953468
Cyrtodactylus baluensis	SPM 06891	GU366079
Cyrtodactylus bansocensis	VFU R.2015.20	MT953469
Cyrtodactylus battalensis	PMNH 2303	KC151984
Cyrtodactylus batucolus	LSUHC 8933	JQ889178
Cyrtodactylus bayinnyiensis	LSUHC 13265	MH198647
Cyrtodactylus bichnganae	UNS 0473	MF169953
Cyrtodactylus bidoupimontis	VNMN 03375	MT953470
Cyrtodactylus bintangrendah	LSUHC 9984	JX519487
Cyrtodactylus bintangtinggi	LSUHC 9006	JX519494
Cyrtodactylus bokorensis	LSUHC 8542	KT013147
Cyrtodactylus boreoclivus	ABTC47722	JQ820307
Cyrtodactylus brevidactylus	CAS214104	JX440527
Cyrtodactylus brevipalmatus	USMHC 2555	MT953472
Cyrtodactylus bugiamapensis	IEBR A.2011.3B	MT953473
Cyrtodactylus calamei	NUOL R-2105.22	MT953474
Cyrtodactylus caovansungi	UNS 0304	MF169954
Cyrtodactylus capreoloides	SAMA R66092	JQ820310

Cyrtodactylus cardamomensis	FMNH 263345	KT013115
Cyrtodactylus cattienensis	UNS 0389	MF169956
Cyrtodactylus cavernicolus	LSUHC4056	JX440528
Cyrtodactylus cf. *agamensis*	ENS 19694	MH248907
Cyrtodactylus cf. *fasciolatum*	CES09/1196	KM255172
Cyrtodactylus cf. *fasciolatum*	CES11/1257	KM255185
Cyrtodactylus cf. *serratus*	SAMA R62635	JQ820297
Cyrtodactylus cf. *peciosus*	CES09/1249	KM878629
Cyrtodactylus chamba	CES11/1291	KM255191
Cyrtodactylus chanhomeae	CUMZ2003.62	JX440529
Cyrtodactylus chaunghanakwaensis	LSUHC13299	MH198644
Cyrtodactylus chauquangensis	NA2016.1	MT953475
Cyrtodactylus chrysopylos	LSUHC 13901	MH764601
Cyrtodactylus collegalensis	CES09/1403	KX632365
Cyrtodactylus condorensis	LSUHC 8587	KT013196
Cyrtodactylus consobrinus	FMNH:230113	GU550725
Cyrtodactylus cucdongensis	UNS 0544	MF169959
Cyrtodactylus cucphuongensis	CP 17.02	MT953477
Cyrtodactylus dammathetensis	LSUHC 12863	MF872277
Cyrtodactylus darmandvillei	WAMR 98719	KU232618
Cyrtodactylus dati	LSUHC 11416	KT013104
Cyrtodactylus dattkyaik	AP018116	MN534902
Cyrtodactylus deccanensis	CES09/1112	KM878615
Cyrtodactylus durio	LSUHC 9725	KU893159
Cyrtodactylus eisenmanae	LSUHC 8598	JX440534
Cyrtodactylus elok	LSUHC 6471	JQ889180
Cyrtodactylus epiroticus	BPBM:39342	KT363952
Cyrtodactylus equestris	AMS R135520	KT835458
Cyrtodactylus evanquahi	BYU 53435	MN586889
Cyrtodactylus fasciolatus	CES11/1337	KM255184
Cyrtodactylus gansi	CAS222412	JX440537
Cyrtodactylus gialaiensis	VNUF R.2017.1	MT953479
Cyrtodactylus grismeri	LSUHC8638	JX440538
Cyrtodactylus guakanthanensis	LSUHC 11322	KU253576
Cyrtodactylus gubaot	KU:309336	GU550779
Cyrtodactylus gubernatoris	CES10/1235	KM255204
Cyrtodactylus gunungsenyumensis	LSUHC 12200	KU253584
Cyrtodactylus guwahatiensis	CES09/1127	KM255194
Cyrtodactylus hidupselamanya	LSUHC:12173	KX011420
Cyrtodactylus himalayanus	CES11/1317	KM255187

Cyrtodactylus hinnamnoensis	VNUF R.2015.3	MT953480
Cyrtodactylus hontreensis	LSUHC8583	JX440539
Cyrtodactylus hoskini	Cyho.GC4	KT363931
Cyrtodactylus huongsonensis	IEBR A.2011.3A	MT953481
Cyrtodactylus huynhi	UNS 0413	MF169963
Cyrtodactylus ingeri_BRK702	BRK702	MN884158
Cyrtodactylus interdigitalis	FMNH:255454	JQ889181
Cyrtodactylus intermedius	LSUHC 8490	KT013138
Cyrtodactylus inthanon	AA06195	XXXXX
Cyrtodactylus irregularis	FMNH:HERP:258697	JX041341
Cyrtodactylus jaegeri	NUOL R.2013.1	MT953482
Cyrtodactylus jaintiaensis	CES10/1228	KM255195
Cyrtodactylus jambangan	KU:314835	GU366102
Cyrtodactylus jarakensis	LSUHC 8990	XXXXX
Cyrtodactylus jarujini	FMNH255472	JX440541
Cyrtodactylus jelawangensis	LSUHC 11062	KJ659852
Cyrtodactylus jellesmae	MVZ239337	JX440542
Cyrtodactylus jeyporensis	CES09/1356	KM878616
Cyrtodactylus kaziriangaensis	CES09/1128	KM255170
Cyrtodactylus khasiensis	CES10/1229	KM255188
Cyrtodactylus kimberleyensis	WAM R164144	JX440544
Cyrtodactylus kingsadai	IEBR A.2013.3	MT953483
Cyrtodactylus klugei	BPBM:1974	HQ401197
Cyrtodactylus laangensis	LSUHC 8770	KT013158
Cyrtodactylus langkawiensis	LSUHC:9120	JX519502
Cyrtodactylus lateralis	MZB:13174	KU893162
Cyrtodactylus lawderanus	CES11/1276	KM255190
Cyrtodactylus leegrismeri	UNS 0449	KT013201
Cyrtodactylus lekaguli	ZMKU<THA>:R00720	KX011425
Cyrtodactylus lenggongensis	LSUHC:9975	JX519488
Cyrtodactylus lenya	USNM:Herp:587788	KY041653
Cyrtodactylus limajalur	CAS262946	MK477177
Cyrtodactylus linnoensis	LSUHC 12825	MF872295
Cyrtodactylus linnwayensis	LSUHC 12984	MF872288
Cyrtodactylus lomyenensis	UNS 0534	MF169966
Cyrtodactylus loriae	ABTC49742	JQ820299
Cyrtodactylus louisiadensis	BPBM:19741	HQ401190
Cyrtodactylus macrotuberculatus	LSUHC:10037	JX519519
Cyrtodactylus majulah	ZRC:26951	JX988529
Cyrtodactylus malayanus	RMBR:00867	GU550733

Cyrtodactylus marmoratus	TNHC59549	JX440546
Cyrtodactylus martini	UNS 0471	MF169968
Cyrtodactylus mcdonaldi	QM:QMJ87078	HQ401139
Cyrtodactylus medioclivus	AMS R122411	JQ820294
Cyrtodactylus meersi	LSUHC 13455	MH624104
Cyrtodactylus metropolis	LSUHC 11344	KU253578
Cyrtodactylus mimikanus	MZB 5343	JQ820316
Cyrtodactylus mombergi	MN059869	LSUHC 14591
Cyrtodactylus montanus	CES10/1211	KM255200
Cyrtodactylus muluensis	CAS262981	MK477167
Cyrtodactylus murua	BPBM:37342	KT363953
Cyrtodactylus myaleiktaung	LSUHC 13948	MH764589
Cyrtodactylus nagalandensis	CES10/1233	KM255199
Cyrtodactylus naungkayaingensis	LSUHC13207	MH198664
Cyrtodactylus nebulosus	CES09/1351	KM878618
Cyrtodactylus nigriocularis	VNMN2184	XXXXX
Cyrtodactylus novaeguineae	MS:R135520	KT363956
Cyrtodactylus nyinyikyawi	CAS:226139	MH624118
Cyrtodactylus oldhami	MS585	MF872302
Cyrtodactylus pageli	ZFMK 91827	XXXXX
Cyrtodactylus pantiensis	LSUHC 8906	JQ889185
Cyrtodactylus papuensis	SAMA R62652	JQ820320
Cyrtodactylus payacola	JQ889190	JQ889190
Cyrtodactylus payarhtanensis	USNM:Herp:587408	KY041654
Cyrtodactylus peguensis	LSUHC 13454	MH756190
Cyrtodactylus petani	MZBLace 11706	KU232620
Cyrtodactylus pharbaungensis	BYU:52215	MF872303
Cyrtodactylus philippinicus	KU:303842	GU550825
Cyrtodactylus phongnhakebangensis	UNS 0347	MF169970
Cyrtodactylus phuocbinhensis	KH-Res041	XXXXX
Cyrtodactylus phuquocensis	UNS 0273	MF169971
Cyrtodactylus pinlaungensis	LSUHC 14279	MN030634
Cyrtodactylus pronarus	HQ401152	HQ401152
Cyrtodactylus psarops	UTA:61580	KR921705
Cyrtodactylus pseudoquadrivirgatus	UNS 0249	MF169972
Cyrtodactylus pubisulcus	LSUHC4069	JX440551
Cyrtodactylus puhuensis	ND 01.15	XXXXX
Cyrtodactylus pulchellus	LSUHC 6729	MF169974
Cyrtodactylus pyadalinensis	CAS:226142	MH624105
Cyrtodactylus pyinyaungensis	BYU:52234	MF872307

Cyrtodactylus quadrivirgatus	LSUHC 4813	MF169975
Cyrtodactylus redimiculus	PNM:1468	GU550738
Cyrtodactylus rex	SAMA R67637	KT835460
Cyrtodactylus rishivalleyensis	CES09/1452	KX698081
Cyrtodactylus robustus	BPBM19731	JX440554
Cyrtodactylus rosichonariefi	MZB:Lace12133	KP256188
Cyrtodactylus rubidus	CES13/1445	KM255203
Cyrtodactylus russelli	CAS226137	JX440555
Cyrtodactylus sadanensis	LSUHC 12853	MF872324
Cyrtodactylus sadansinensis	BYU:52220	MF872325
Cyrtodactylus sadleiri	AMS152676	MH105038
Cyrtodactylus saiyok	MS484	MF872308
Cyrtodactylus salomonensis	AMS134930	JX440556
Cyrtodactylus sanpelensis	LSUHC 12889	MF872345
Cyrtodactylus semenanjungensis	LSUHC 8900	JQ889177
Cyrtodactylus semicinctus	ENS 14966	KR921713
Cyrtodactylus septentrionalis	BNHS 1989	MH971164
Cyrtodactylus septimontium	NAP 05322	MH940233
Cyrtodactylus seribuatensis	LSUHC 6348	JX440557
Cyrtodactylus sermowaiensis	ABTC47761	JQ820296
Cyrtodactylus sharkari	LSUHC 11022	KJ659853
Cyrtodactylus shwetaungorum	LSUHC 12898	MF872353
Cyrtodactylus sinyineensis	LSUHC 12836	MF872355
Cyrtodactylus slowinskii	CAS210205	JX440559
Cyrtodactylus sommerladi	VNUF R.2013.22	MT953490
Cyrtodactylus soni	IEBR R.2016.4	MT953491
Cyrtodactylus sonlaensis	IEBR A.2017.1	MT953492
Cyrtodactylus soudthichaki	VFU R.2015.18	MT953493
Cyrtodactylus sp_11_IA_2014	CES09/1197	KM255181
Cyrtodactylus sp_2_IA_2014	CES10/1459	KM255192
Cyrtodactylus sp_2_PLW_2015	LSUHC 11414	KT013199
Cyrtodactylus sp_3_IA_2014	CES10/1465	KM255193
Cyrtodactylus sp_6_IA_2014	CES10/1464	KM255196
Cyrtodactylus sp_Borneo	BRK623	MF706373
Cyrtodactylus sp_CDS_2010a	RMBR:00321	GU550728
Cyrtodactylus sp_Gobe_Ridg	SAMA R62654	JQ820322
Cyrtodactylus sp_Huon_Peninsula	SAMA R65954	JQ820318
Cyrtodactylus sp_KAC_2015a	ENS:15784	KR921689
Cyrtodactylus sp_KAC_2015a	ENS:15813	KR921697
Cyrtodactylus sp_KAC_2015a	ENS:13779	KR921699

Cyrtodactylus sp_KAC_2015a	MZB:9676	KR921700
Cyrtodactylus sp_KAC_2015a	ENS:14656	KR921711
Cyrtodactylus sp_KAC_2015a	ENS:15575	KR921720
Cyrtodactylus sp_Kai_Islands	XXXXX	MF706380
Cyrtodactylus sp_Timor	USNM579045	JX440560
Cyrtodactylus sp_WAMR_107499	WAMR 107499	KU232623
Cyrtodactylus sp_WAMR_109089	WAMR 109089	KU232625
Cyrtodactylus sp_WAMR_109893	WAMR 109893	KU232621
Cyrtodactylus sp_WAMR_112000	WAMR 112000	KU232624
Cyrtodactylus spMH248914	ENS 18711	MH248914
Cyrtodactylus spMH248938	ENS 13779	MH248938
Cyrtodactylus spMH248941	ENS 13748	MH248941
Cyrtodactylus spinosus	MZB 7024	MT953494
Cyrtodactylus srilekhae	CES09/1536	KX698084
Cyrtodactylus sumuroi	KU:310798	GU550772
Cyrtodactylus sworderi	LSUHC 7685	JQ889189
Cyrtodactylus takouensis	UNS 0486	MF169978
Cyrtodactylus tanim	SJR1470	MF706378
Cyrtodactylus taungwineensis	LSUHC 14115	MN534925
Cyrtodactylus tautbatorum	KU:309322	GU550752
Cyrtodactylus taybacensis	IEBR4379	MT953495
Cyrtodactylus tebuensis	LSUHC 10903	JX988528
Cyrtodactylus teyniei_1	KM2012.14	MT953496
Cyrtodactylus thirakhupti	XXXXX	AP018115
Cyrtodactylus thylacodactylus	LSUHC 9319	KT013163
Cyrtodactylus tibetanus	MVZ233251	JX440561
Cyrtodactylus tigroides	IRSNB2380	JX440562
Cyrtodactylus timur	KJ659857	KJ659857
Cyrtodactylus tiomanensis	JAM 1879	GU550734
Cyrtodactylus triedrus	ADS35	JX440522
Cyrtodactylus trilatofasciatus	JX519530	JX519530
Cyrtodactylus tripartitus	JQ820317	JQ820317
Cyrtodactylus triperanensis	CES10/1216	KM255183
Cyrtodactylus tuberculatus	QM:J88555	KT363943
Cyrtodactylus varadgirii	BNHS 2099	KX632368
Cyrtodactylus vilaphongi	IEBR A.2013.103	MT953497
Cyrtodactylus wayakonei	ZFMK91016	MT953498
Cyrtodactylus welpyanensis	LSUHC 12785	MF872360
Cyrtodactylus yangbayensis	LSUHC 11407	KT013202
Cyrtodactylus yathepyanensis	BYU:52228	MF872363

Cyrtodactylus yoshii	JX440565	JX440565
Cyrtodactylus ywanganensis	LSUHC 13712	MH607610
Cyrtodactylus zebriacus	XXXXX	MF100157
Cyrtodactylus zugi	MZB 5575	JQ820306

Supplemental Table 3. Results from BLAST of female-specific *Cyrtodactylus pharbaungensis* RAD contigs that were searched against *Hemidactylus turcicus* transcripts and then to chicken (*Gallus gallus*) genes. Nineteen of 38 matches are on chicken chromosome 10.

Ensembl Gene ID	Gene	Chicken chromosome	E Value	Query	*C. pharbaugensis* RAD ID
ENSGALG00000037962	C1H21ORF59	1	0	Hemidactylus_CL2004Contig1_1_CU059	Cp543
ENSGALG00000014526	TPI1	1	0	Hemidactylus_TRINITY_DN24306_c7_g3_i4_1_TPIS	Cp263;Cp109;Cp169;Cp175;Cp442
ENSGALG00000012923	CDH9	2	0	Hemidactylus_CL1601Contig1_1_CADH9	Cp302
ENSGALG00000010835	DTX4	5	0	Hemidactylus_CL44Contig2_1_DTX4	Cp388;Cp490;Cp54;Cp642
ENSGALG00000011639		5	0	Hemidactylus_CL645Contig1_1_C170B	Cp361
ENSGALG00000023135	SRP54	5	0	Hemidactylus_CL889Contig1_1_SRP54	Cp146
ENSGALG00000002638		6	0	Hemidactylus_CL1Contig109_1_FSDH	Cp323
ENSGALG00000037238	PCCB	9	0	Hemidactylus_CL251Contig1_2_PCCB	Cp289
ENSGALG00000003322	TBC1D2B	10	0	Hemidactylus_TRINITY_DN21669_c0_g2_i2_1_TBD2B	Cp163
ENSGALG00000043828	HEXA	10	0	Hemidactylus_TRINITY_DN22594_c0_g2_i1_1_HEXA	Cp447
ENSGALG00000004212	ADAM10	10	0	Hemidactylus_TRINITY_DN22900_c0_g2_i3_1_ADA10	Cp256
ENSGALG00000002150	CPEB1	10	0	Hemidactylus_TRINITY_DN23138_c0_g1_i3_1_CPE1A	Cp168
ENSGALG00000003628	TLN2	10	0	Hemidactylus_TRINITY_DN24076_c6_g2_i3_1_TLN2	Cp201
ENSGALG00000001992	PKM	10	0	Hemidactylus_TRINITY_DN24916_c2_g2_i14_1_KPYM	Cp467;Cp539
ENSGALG00000001798	BBS4	10	0	Hemidactylus_TRINITY_DN25169_c9_g1_i4_1_BBS4	Cp604
ENSGALG00000003906	MPHOSPH10	10	0	Hemidactylus_TRINITY_DN25243_c7_g5_i2_1_MPP10	Cp209
ENSGALG00000041362	SECISBP2L	10	0	Hemidactylus_TRINITY_DN25455_c4_g1_i3_1_SBP2L	Cp301
ENSGALG00000001376	ARID3B	10	0	Hemidactylus_TRINITY_DN25732_c4_g1_i1_1_ARI3B	Cp82
ENSGALG00000004347	NEDD4	10	0	Hemidactylus_TRINITY_DN25733_c6_g2_i2_1_NEDD4	Cp357
ENSGALG00000031448	MAPK6	10	0	Hemidactylus_TRINITY_DN25818_c4_g1_i3_1_MK06	Cp412
ENSGALG00000041896	RFX7	10	0	Hemidactylus_TRINITY_DN25991_c1_g2_i2_3_RFX7	Cp146
ENSGALG00000039307	GNB5	10	0	Hemidactylus_TRINITY_DN26409_c30_g2_i3_1_GNB5	Cp191
ENSGALG00000013225	VAT1L	11	0	Hemidactylus_CL3907Contig1_1_VAT1L	Cp326
ENSGALG00000001945	RFT1	12	0	Hemidactylus_TRINITY_DN23547_c10_g1_i3_2_RFT1	Cp231
ENSGALG00000039288	RBM10	12	0	Hemidactylus_TRINITY_DN25589_c7_g1_i8_1_RBM5	Cp171
ENSGALG00000035080	HARS	13	0	Hemidactylus_TRINITY_DN25226_c1_g6_i2_1_SYHC	Cp284
ENSGALG00000033989	SLC39A14	22	0	Hemidactylus_TRINITY_DN26324_c9_g7_i1_1_S39AE	Cp569
ENSGALG00000003733	LIFR	Z	0	Hemidactylus_TRINITY_DN26126_c1_g3_i4_1_LIFR	Cp585
ENSGALG00000003505	LACTB	10	2.67E-175	Hemidactylus_TRINITY_DN7417_c0_g1_i1_1_LACTB	Cp112
ENSGALG00000046358	FBLN7	3	4.38E-171	Hemidactylus_TRINITY_DN25782_c7_g16_i1_1_FBLN7	Cp635
ENSGALG00000002093	NR2E3	10	3.11E-151	Hemidactylus_TRINITY_DN18395_c0_g2_i1_1_NR2E3	Cp613
ENSGALG00000041878	AQP5	33	1.98E-150	Hemidactylus_CL723Contig1_1_AQP5	Cp497
ENSGALG00000049218	DYX1C1	10	1.57E-116	Hemidactylus_TRINITY_DN23842_c3_g1_i2_1_DAAF4	Cp252
ENSGALG00000011041	DNAJC6	8	1.32E-110	Hemidactylus_CL1769Contig1_2	Cp183
ENSGALG00000007084	DUSP12	1	3.28E-84	Hemidactylus_CL2335Contig1_2_DUS12	Cp417
ENSGALG00000001470	STOML1	10	4.57E-77	Hemidactylus_TRINITY_DN24180_c0_g1_i3_1_STML1	Cp71
ENSGALG00000014768	SGTB	Z	5.35E-64	Hemidactylus_CL4193Contig1_1	Cp391
ENSGALG00000035202	AP3B2	10	1.56E-58	Hemidactylus_TRINITY_DN25210_c6_g1_i2_1	Cp211

Supplemental Table 4. Results from BLAST of male-specific *Cyrtodactylus chaunghanakwaensis* RAD contigs that were searched against *Hemidactylus turcicus* transcripts and then to chicken (*Gallus gallus*) genes.

Ensembl Gene ID	Gene	Chicken chromosome	E Value	Query	*C. chaunghanakwaensis* RAD ID
ENSGALG00000039208	PHF2	12	0	Hemidactylus_CL4739Contig1_1_PHF2	Cc33
ENSGALG00000049176		1	2.63E-97	Hemidactylus_TRINITY_DN24874_c0_g1_i13_1_CE290	Cc103
